# A facile one-pot oxidation-assisted dealloying protocol to massively synthesize monolithic core-shell architectured nanoporous copper@cuprous oxide nanonetworks for photodegradation of methyl orange

**DOI:** 10.1038/srep36084

**Published:** 2016-11-10

**Authors:** Wenbo Liu, Long Chen, Xin Dong, Jiazhen Yan, Ning Li, Sanqiang Shi, Shichao Zhang

**Affiliations:** 1 School of Manufacturing Science and Engineering, Sichuan University, Chengdu 610065, China; 2 Department of Mechanical Engineering, The Hong Kong Polytechnic University, Hung Hom, Kowloon, Hong Kong; 3 School of Materials Science and Engineering, Beihang University, Beijing 100191, China

## Abstract

In this report, a facile and effective one-pot oxidation-assisted dealloying protocol has been developed to massively synthesize monolithic core-shell architectured nanoporous copper@cuprous oxide nanonetworks (C-S NPC@Cu_2_O NNs) by chemical dealloying of melt-spun Al 37 at.% Cu alloy in an oxygen-rich alkaline solution at room temperature, which possesses superior photocatalytic activity towards photodegradation of methyl orange (MO). The experimental results show that the as-prepared nanocomposite exhibits an open, bicontinuous interpenetrating ligament-pore structure with length scales of 20 ± 5 nm, in which the ligaments comprising Cu and Cu_2_O are typical of core-shell architecture with uniform shell thickness of ca. 3.5 nm. The photodegradation experiments of C-S NPC@Cu_2_O NNs show their superior photocatalytic activities for the MO degradation under visible light irradiation with degradation rate as high as 6.67 mg min^−1^ g_cat_^−1^, which is a diffusion-controlled kinetic process in essence in light of the good linear correlation between photodegradation ratio and square root of irradiation time. The excellent photocatalytic activity can be ascribed to the synergistic effects between unique core-shell architecture and 3D nanoporous network with high specific surface area and fast mass transfer channel, indicating that the C-S NPC@Cu_2_O NNs will be a promising candidate for photocatalysts of MO degradation.

Nowadays, more than 700,000 tons of dye-stuff are produced every year around the world to meet the growing demand of textile industry, in which at least one fifth of them are directly discharged into sea or river without effective treatments by factories with extensive mode of production, especially in less developed and developing countries^1^,^2^. This is fatally detrimental to the environment, aquatic creatures and even humanity itself. Therefore, removal of dyes from sea or river water is a key. Among these dyes, synthetic azo colorants are quite hard to be degraded due to their complex chemical structures^3^. The conventional methods to process wastewater containing azo colorants include coagulation-flocculation, membrane separation, physisorption, etc., in which adsorption is a convenient and effective treatment approach due to its simplicity, low cost, and ease of operation^4^,^5^,^6^,^7^. However, one of its obvious drawbacks is that dye molecules just can transfer from liquid to solid in essence, which cannot be radically eliminated, and thereby contamination still exists just in an alternative way^8^.

Photocatalysis, as a new and efficient methodology, has attracted much attention to process wastewater containing azo colorants in the recent years. Semiconductor-based photocatalysts have been extensively studied, such as titanium dioxide (TiO_2_), zinc oxide (ZnO), and cuprous oxide (Cu_2_O)^9^,^10^,^11^,^12^,^13^. Among them, Cu_2_O, a p-type semiconductor, has been regarded as a more promising photocatalyst to degrade azo colorants due to its narrower band gap (2.0–2.2 eV) than those of TiO_2_ or ZnO (3.0–3.2 eV), which can absorb a large part of visible light (λ < 620 nm) effectively^9^,^14^. In the past decades, Cu_2_O photocatalysts with various micro/nanostructures has been prepared through different ways including electrolysis, reduction of Cu^2+^ salts, and hydrothermal/solvothermal methods, etc^15^,^16^,^17^. When exposed to visible light, Cu_2_O semiconductor can be excited to produce photo-generated electrons and holes, which can initiate a series of photodegradation reactions. However, photo-generated electrons readily tend to recombine with photoholes in Cu_2_O-based photocatalysts, and thus limiting their photocatalytic activities severely^18^. One effective way to solve this issue is to construct the heterojunction of Cu/Cu_2_O to enhance the photocatalytic properties of Cu_2_O-based semiconductors since Cu substrates can promptly transfer photoelectrons and avoid the recombination of electron-hole pairs^19^.

Nanoporous metals (NPMs), as novel and superior substrate materials, have recently attracted great interest for their wide applications in catalysis, sensors, actuators, and so forth, owing to their unique physical, chemical, and mechanical properties associated with their high surface-to-volume ratio and low densities^20^,^21^,^22^,^23^,^24^,^25^,^26^,^27^. Dealloying has been proved to be a simple and effective approach to generate various kinds of NPMs via selective dissolution of one or more active elements out of suitable precursors, eventually leading to a 3D, biocontinuous interpenetrating ligament-pore nanonetwork^28^,^29^,^30^,^31^,^32^. Recently, NPMs have been considered as good substrate materials of catalysts with superior electro/photocatalytic activities through surface modification because of their monolithic morphology, large specific surface areas and excellent electrical conductivity. For example, Ding *et al*.^33^ reported that Pt-modified nanoporous gold possesses high catalytic performance towards alcohol-species electrooxidation through dealloying and epitaxial growth techniques. Kou *et al*.^34^ synthesized nanoporous Cu-supported Cu_2_O photocatalysts with good catalytic activity using a two-step route involving dealloying and surface oxidation. However, these multi-step approaches cannot be high-efficient enough for practical applications, which remarkably prolongs the production time and increases the cost; compared to it, one-pot synthesis protocol could have evident advantages of simplicity, economy, and being applicable for large-scale production. Thus, avoiding multi-step manipulation and achieving facile one-pot mass synthesis of NPM@metal oxide composites are crucial for their promisingly industrial applications, which urgently needs to be investigated.

It is well recognized that dealloying process normally should be carried out in an oxygen-purged corrosive medium, such as hydrochloric acid or sodium hydroxide dilute solution, in order to obtain absolute nanoporous metals avoiding the occurrence of spontaneous side reactions, especially for that between dissolved active oxygen and metal adatoms on alloy/solution interfaces^35^,^36^,^37^. Instead of it, in this case, we just could make use of the spontaneous side reaction skillfully to achieve the one-pot mass synthesis of NPM@metal oxide composites by dealloying of suitable precursor alloys in an oxygen-rich corrosive environment, especially for nanoporous Cu@Cu_2_O photocatalysts for degradation of MO, one of the most common azo colorants in textile, printing, paper manufacturing, pharmaceutical and food industries^38^.

To test the feasibility of idea, the designed bi-phase Al 37 at.% Cu precursor alloy has been taken as a typical instance to fabricate the monolithic nanoporous Cu@Cu_2_O nanocomposites for photodegradation of MO through one-pot oxidation-assisted dealloying route, as schematically illustrated in Fig. 1a. The experimental results show that the monolithic nanoporous Cu@Cu_2_O nanonetworks with core-shell architectures can be obtained on a large scale by the facile one-pot chemical dealloying in an oxygen-rich alkaline solution under free corrosion condition, which exhibits excellent photocatalytic activities towards the degradation of MO. Besides, the formation and photodegradation mechanisms are discussed in detail.

## Results and Discussion

Figure 2 shows the XRD patterns of the initial Al 37 at.% Cu alloy ribbons and their as-dealloyed nanocomposites upon dealloying in the oxygen-rich NaOH solution, respectively. The filled circles, squares, triangles and stars in Fig. 2 stand for Al_2_Cu, AlCu, Cu and Cu_2_O, respectively. The initial Al 37 at.% Cu alloy is composed of two phases: Al_2_Cu and AlCu, in which the Al_2_Cu is predominant in the alloy (Fig. 2a). Upon the dealloying in the oxygen-rich NaOH solution, three major diffraction peaks (2θ = 43.3, 50.4 and 74.1°) can be found in the XRD pattern, assigning to the (111), (200) and (220) reflections of face centered cubic (f.c.c.) Cu, respectively. Moreover, there are two minor diffraction peaks (2θ = 36.5 and 61.5°) which can be determined to be Cu_2_O phase, corresponding to its (111) and (220) reflections respectively. It should be noted that other diffraction peaks of Cu_2_O at 42.4° and 72.7° cannot be readily discerned due to the occurrence of overlaps with (111)_Cu_ and (220)_Cu_ reflections. Thus, the as-prepared nanocomposites are composed of Cu and Cu_2_O phases, in which Cu is dominant obviously based on their relative peak intensities (Fig. 2b).

The plane view of the as-prepared nanocomposites from Al 37 at.% Cu alloy shows that a uniform porous structure can be obtained upon dealloying in the oxygen-rich NaOH solution and one typical SEM image is shown in Fig. 3a. Clearly, the surface morphology at a high magnification exhibits an open, bicontinuous interpenetrating ligament-pore structure with length scales of 20 ± 5 nm (Fig. 3b). The section view of the nanocomposites displays the uniform pores continuously penetrate the whole ribbons, as presented in Fig. 3c. The fracture surface of the samples also exhibits an open, bicontinuous interpenetrating ligament-pore structure, suggesting that the as-made nanoporous structure is three-dimensional (Fig. 3d). Additionally, EDX analysis shows both Cu and O can be identified simultaneously from the surface and section of the nanocomposites and nearly all of Al was etched away during dealloying by the NaOH solution (Fig. 3e,f), indicating that Cu and Cu_2_O phases coexist in the whole samples in combination with the XRD results. In contrast, NPG (by dealloying of Ag-Au alloys) normally contains some residual at.% Ag but no extra O, which is expected to be trapped inside the Au ligaments based upon the dealloying mechanism and cannot be removed but asymptotically reaches a limit at exhaustively long etching times (up to 100 h)^39^,^40^.

TEM observation further verifies the uniform porous structure of as-prepared nanocomposites, which is well consistent with the SEM results and one typical TEM bright-field image is displayed in Fig. 4a. It is obvious that a thin coating can be observed on the ligament surface of porous structure, constituting a typical core-shell nanoarchitecture. The TEM image in a higher magnification shows the thickness of uniform shell layer is ca. 3.5 nm, as marked by double arrows in Fig. 4b. Moreover, different lattice fringes can be seen clearly in the HRTEM image as well as the interplanar spacings of 0.182 nm and 0.245 nm correspond to the (002) reflection of Cu phase and the (111) reflection of Cu_2_O phase, respectively. A clear boundary between Cu core and Cu_2_O shell can be distinguished based on their different lattice orientations. It should be noted that lattice fringes from Cu and Cu_2_O extending throughout the whole ligament and thin coating indicates their single crystal nature respectively, which is essentially different from the established notion that the crystal lattice orientation is retained during dealloying of Ag-Au alloy solid solutions with the conservation of grain size of master alloys due to the intrinsic disparity in lattice structure between master alloys and products (Al_2_Cu: body centered tetragonal; AlCu: end centered monoclinic; Cu: f.c.c.)^41^,^42^,^43^,^44^. Due to the small misorientation between lattice fringes of Cu (002) and Cu_2_O (111), it can be reasonable to assume that the Cu_2_O shell layer can be formed *in-situ* through epitaxial growth on the Cu core substrates during the dealloying. As a result, the as-prepared monolithic nanoporous Cu@Cu_2_O nanocomposites have perfect core-shell architectures by the facile one-pot oxidation-assisted dealloying route.

Figure 5 shows Tafel polarization curves of single-phase Al_2_Cu and AlCu intermetallics in the oxygen-rich NaOH solution at RT, respectively. It can be found that in the alkaline solution, the difference between free corrosion potentials of single-phase Al_2_Cu and AlCu intermetallics is very large and ca. 578 mV(SCE), indicating that a relatively high electrochemical activity can be obtained for Al_2_Cu in the alkaline solution compared to AlCu.

The specific surface area and of the as-prepared nanocomposites has been evaluated and their mesoporous feature has been further confirmed based upon N_2_ adsorption/desorption experiments, which exhibits a type IV isotherm with the H1 hysteresis loop. Figure 6 shows the N_2_ adsorption/desorption isotherms for C-S NPC@Cu_2_O NNs by dealloying of the melt-spun Al 37 at.% Cu alloy in the oxygen-rich NaOH solution at RT. The Brunauer-Emmett-Teller (BET) surface area and total pore volume of the resultant nanocomposites are much high and have been determined to be 32.4 ± 0.1 m^2^ g^−1^ and 0.19 cm^3^ g^−1^ respectively, which would be especially beneficial for photocatalysis applications due to the more active adsorption sites provided for the high-efficient photodegradation process. Moreover, the corresponding pore size distribution curve (inset in Fig. 6) obtained by the Barrett-Joyner-Halenda (BJH) method using the desorption branch of the isotherm shows a sharp peak centered at 19.61 nm, in good coincidence with the pore sizes measured statistically from the SEM and TEM images.

The formation mechanism of the nanocomposites can be rationalized as follows. As well-known, the dealloying process started with selective dissolution of base metal atoms from the outermost alloy surface, leaving behind noble metal atoms that diffused along alloy/solution interfaces and agglomerated into the 3D, bicontinuous interpenetrating porous network^31^,^32^,^33^. In this case, the initial Al 37 at.% Cu alloy composed of Al_2_Cu and AlCu phases, in which the electrochemical activity of Al_2_Cu is greatly higher than AlCu in the alkaline solution, as indicated in Fig. 5. So the dealloying process is first confined to the Al_2_Cu phase and then spreading out in the AlCu, eventually resulting in the formation of porous structure with uniform pore size distributions. On the other hand, as the corrosive solution is oxygen-rich, the dissolved active oxygen can be captured fast by the Cu adatoms with unsaturated bond and high surface energy on the alloy/electrolyte interface, thus further forming Cu_2_O shell layers on the ligament surfaces of NPC. This is why the monolithic C-S NPC@Cu_2_O NNs can be achieved by the one-pot oxidation-assisted dealloying route. Additionally, the thickness of Cu_2_O shell could be adjusted effectively by changing the dealloying duration.

The photocatalytic performance of the as-prepared nanocomposites was evaluated by photodegradation of MO under visible light irradiation. Figure 7 shows the typical UV-vis adsorption spectra and corresponding irradiation time depedence of photodegradation ratio for photocatalytic degradation of MO by the C-S NPC@Cu_2_O NNs. As can be seen clearly in Fig. 7a, the UV-vis absorption spectra exhibit that the intensity of the characteristic absorbance peak of MO at its maximum absorbance wavelength (λ = 465 nm) decreases with the increase of irradiation time. After 100 min, about 90% of MO was degraded effectively, which can be made out from the corresponding plot of photodegradation ratio *versus* irradiation time in Fig. 7b. The irradiation time depedence curve indicates that the photodegradation ratio gradually increases from 0 up to ~90% with increasing the irradiation time continually, and the degradation rate appears first quick back slow trend based on the change in the slope of curve. Note that the present experimental results from separate samples display a relatively narrow error limit, indicative of favorable test stability and reproducibility.

**Figure 1:**
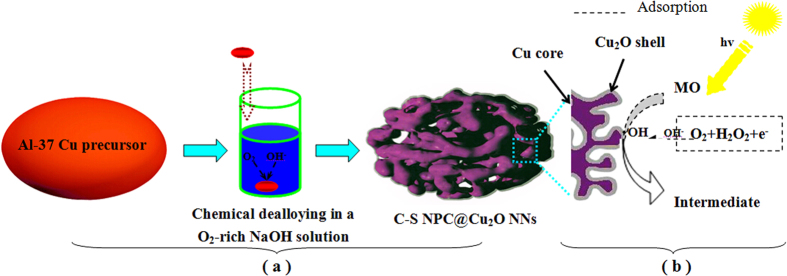
The schematic illustration showing (**a**) the preparation process of the monolithic C-S NPC@Cu_2_O NNs and (**b**) the photodegradation mechanism of MO.

**Figure 2:**
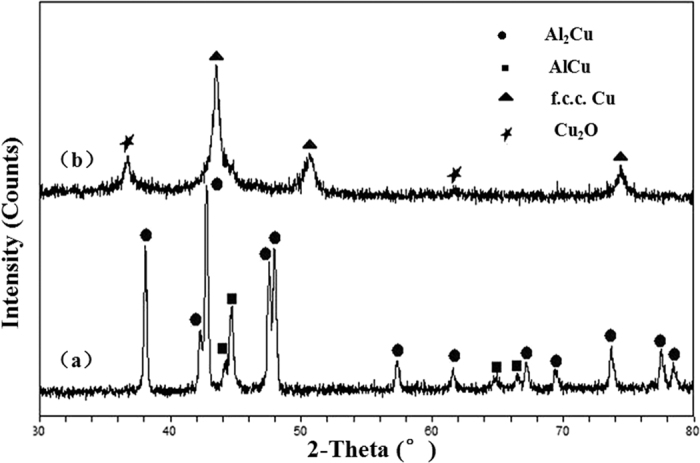
XRD patterns of melt-spun Al 37 at.% Cu alloy ribbons (**a**) before and (**b**) upon dealloying in the oxygen-rich NaOH solution at RT, respectively.

**Figure 3:**
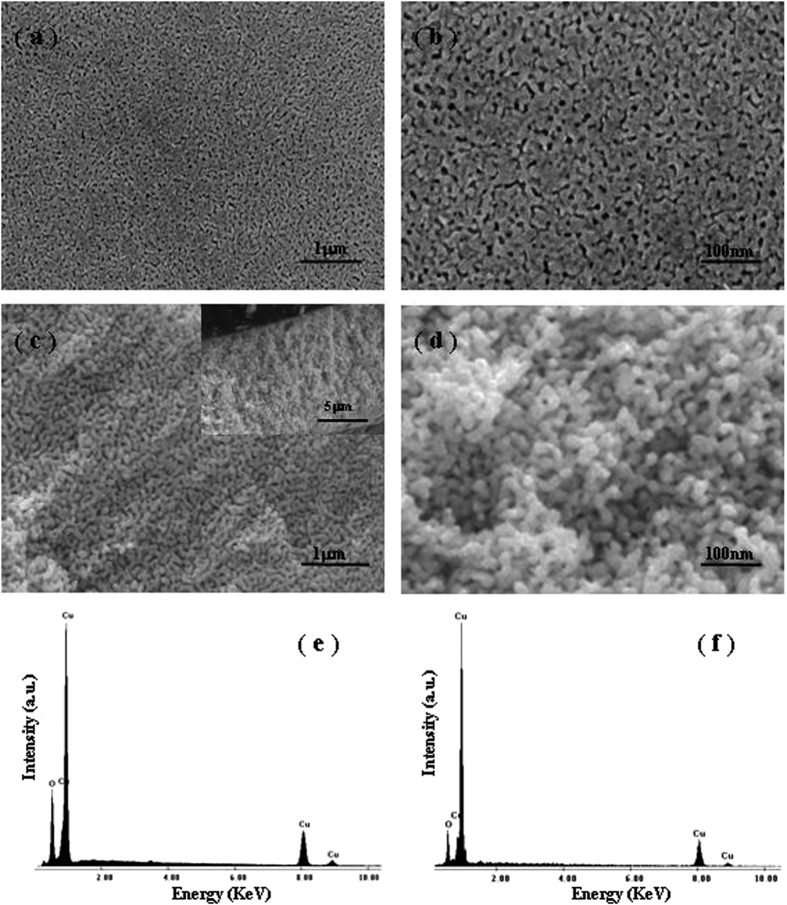
SEM images showing the microstructure of the C-S NPC@Cu_2_O NNs by dealloying of the Al 37 at.% Cu alloy in the oxygen-rich NaOH solution at RT. Parts (**a**,**b**) are the plane views; parts (**c**,**d)** are the section views. Inset in part (**c**) shows the entire section-view image at a lower magnification. (**e**,**f**) Typical EDX spectra showing the chemical compositions of the surface and section of the resultant C-S NPC@Cu_2_O NNs.

**Figure 4:**
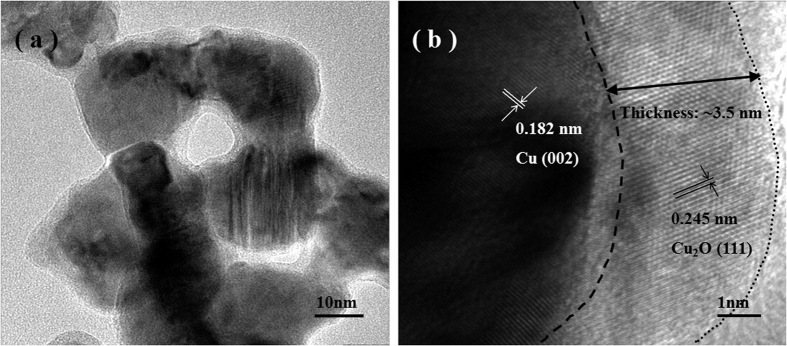
(**a**) TEM image shows the porous structure of C-S NPC@Cu_2_O NNs by dealloying of the Al 37 at.% Cu alloy in the oxygen-rich NaOH solution at RT. (**b**) HRTEM image shows the different lattice fringes on the ligament surface corresponding to Cu core and Cu_2_O shell respectively. Broken line indicates the boundary between Cu core and Cu_2_O shell.

**Figure 5:**
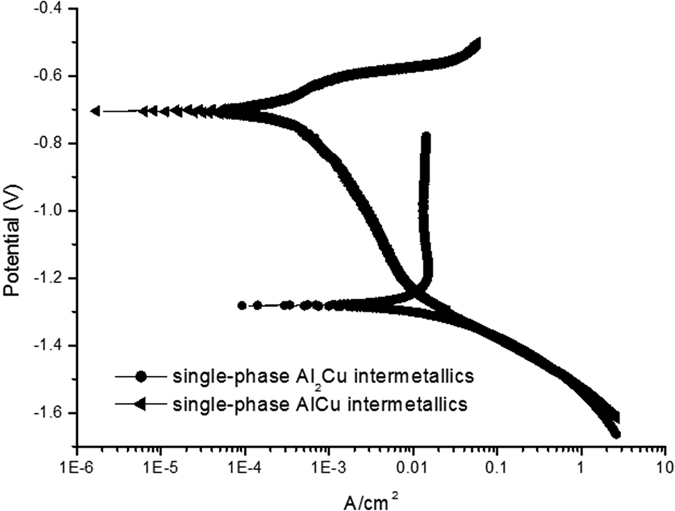
Tafel polarization curves of single-phase Al_2_Cu and AlCu intermetallics in the oxygen-rich NaOH solution at RT.

**Figure 6:**
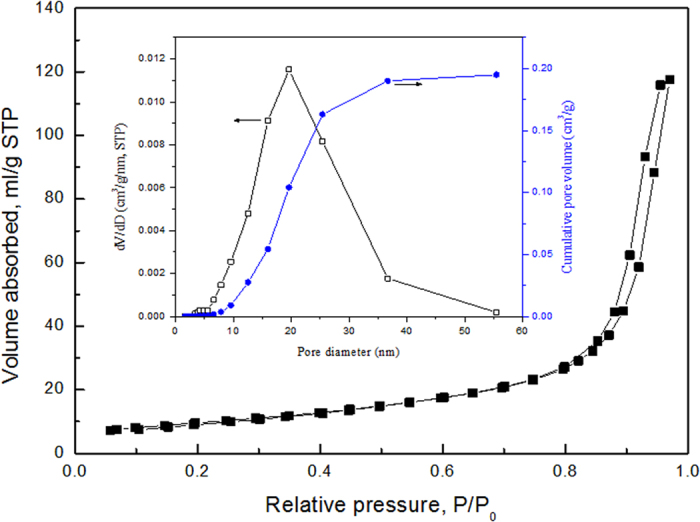
N_2_ isotherm at 77 K for the C-S NPC@Cu_2_O NNs by dealloying of the Al 37 at.% Cu alloy in the 10 wt.% NaOH solution at RT. The inset shows the corresponding pore size distribution and cumulative pore volume.

**Figure 7:**
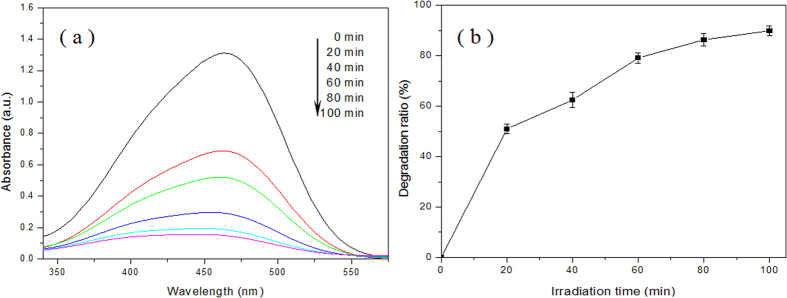
(**a**) UV-vis adsorption spectra and (**b**) irradiation time depedence curve of photodegradation ratio for photocatalytic degradation of MO by the C-S NPC@Cu_2_O NNs.

The MO photodegradation mechanism can be described briefly in Fig. 1b. When exposed to the visible light, the Cu_2_O shell can be excited to produce photo-generated electrons and holes, which can initiate a series of photodegradation reactions. The holes at the valence band can oxidize OH^−^ adsorbed on the photocatalyst surface to produce •OH (hydroxyl radicals) which has a relatively high redox potential (+1.9 V(SHE)) and can oxidize adsorbed MO (+0.94 V(SHE)) effectively^45^,^46^,^47^,^48^. On the other hand, the photo-generated electrons conducted away from holes by Cu can be captured by adsorbed oxygen molecules, leading to the generation of H_2_O_2_, OH^−^, or O_2_^−^, in which O_2_^−^ can further interact with H_2_O_2_ and facilitate the photodegradation of MO^48^,^49^. Finally, MO can be oxidized into intermediates and be desorbed from the surface of Cu_2_O photocatalysts.

**Figure 8:**
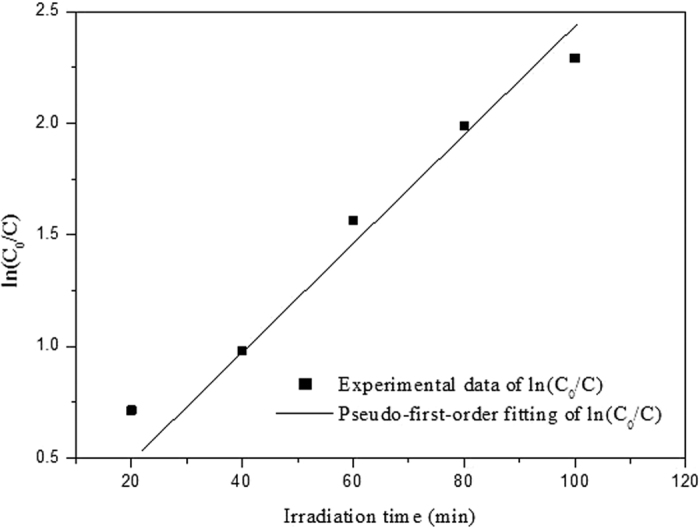
Pseudo-first-order kinetic fitting for photocatalytic degradation of MO by the C-S NPC@Cu_2_O NNs.

It has been well recognized that the MO degradation rate can roughly reflect the degradation efficiency of photocatalysts^34^. The estimated mean value for the MO degradation rate in this case is ~6.67 mg min^−1^ g_cat_^−1^, which are greatly larger than those reported in the literature under similar degradation conditions^19^,^50^,^51^,^52^. The experimental data of photodegradation can be well fitted by pseudo-first-order kinetic model, in which the kinetic equation was used to describe the process as follows^52^:


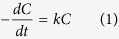


where *C* is the concentration of MO at time *t*, and *k* is the apparent reaction rate constant. By integrating Eqn. (1), we can obtain the linear plot of ln(*C*_0_/*C*) *versus t* as:


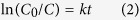


where *C*_*0*_ is the concentration of MO at the irradiation time *t* = 0. Figure 8 shows the pseudo-first-order kinetic fitting plot of ln(*C*_0_/*C*) *versus t* (through origin) for photocatalytic degradation of MO by the C-S NPC@Cu_2_O NNs. It is clear that the good linear relationship can be obtained for the present experimental data. The apparent reaction rate constant can be determined to be 0.024 from its slope, indicative of the faster reaction rate than those obtained in the literature under similar degradation conditions^51^,^52^.

The excellent photocatalytic activity of the as-prepared nanocomposites can be mainly attributed to three aspects as follows: (1) the unique core-shell architecture constituting the ideal heterojunction of Cu/Cu_2_O plays an important part in raising the photocatalytic activity, in which the Cu_2_O shell can be excited to produce photo-generated electrons and holes as well as the Cu core can facilitate the fast transfer of photoelectrons; (2) the high specific surface area of the as-prepared nanocomposites can provide more active adsorption sites for the MO photodegradation process; (3) the 3D bicontinuous nanoporous structure is conducive to the fast transfer of electrolytes and involved diffusion species (such as MO, OH^−^, intermediate, O_2_).

**Figure 9:**
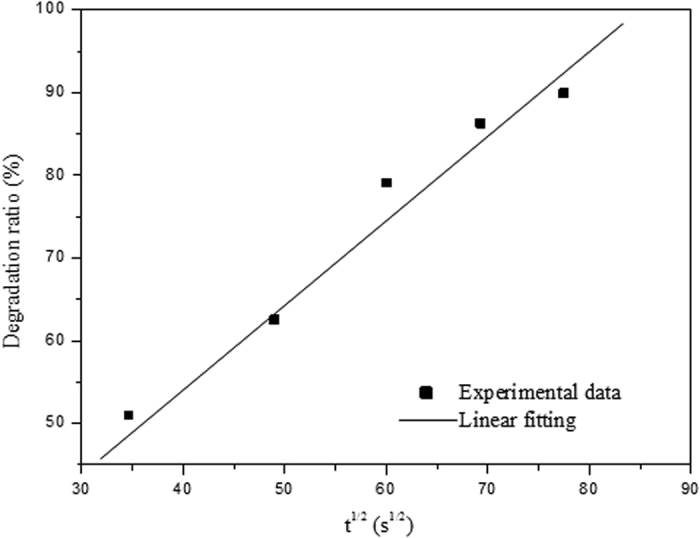
The fitting plot of photodegradation ratio *versus* the square root of irradiation time for photocatalytic degradation of MO by the C-S NPC@Cu_2_O NNs.

Figure 9 shows the fitting plot of photodegradation ratio *versus* the square root of irradiation time for photocatalytic degradation of MO by the C-S NPC@Cu_2_O NNs. Obviously, the good linear correlation can be acquired for the photodegradation process, implying that the MO photodegradation in this case is a diffusion-controlled kinetic process^53^,^54^. As indicated above, adsorption is the key to the photodegradation process, and thus the results further demonstrate that diffusion of the involved species herein plays a crucial role in the adsorption stage. Additionally, the slope of the fitted line can reflect the photodegradation rate and has been determined to be 1.25, indicative of its relatively fast photocatalytic degradation by the present photocatalyst, which is also in good agreement with the estimated value for MO photodegradation rate.

Compared to most of Cu@Cu_2_O photocatalysts with various nanostructures in the literature^19^,^34^,^52^, it is the first to report that the monolithic nanoporous Cu@Cu_2_O photocatalysts with core-shell architectures can be synthesized massively by the facile one-pot oxidation-assisted dealloying route. It needs to be pointed out that the photocatalytic performance of the as-prepared C-S NPC@Cu_2_O NNs could be further improved by optimizing their microstructure (such as surface morphology, ligament/pore sizes and distribution) and nanoporosity which will be one of our focuses in the near future. Based on our present findings, it can be proposed that this effective one-pot oxidation-assisted dealloying protocol can be extended to synthesize other core-shell architectured NPMs@M_x_O_y_ nanocomposites in large as promisingly high-efficient, low-cost photocatalysts towards photodegradation of organic dyes.

## Conclusions

In summary, we present a facile and effective one-pot oxidation-assisted dealloying protocol to massively synthesize the monolithic core-shell architectured nanoporous copper@cuprous oxide nanonetworks by chemical dealloying of melt-spun bi-phase Al 37 at.% Cu alloy in the oxygen-rich NaOH solution at RT. The as-prepared C-S NPC@Cu_2_O NNs exhibits an open, bicontinuous interpenetrating porous network with ligament/pore sizes of 20 ± 5 nm and BET surface area of 32.4 ± 0.1 m^2^ g^−1^, in which the ligaments (namely pore walls) composed of Cu and Cu_2_O phases are characteristic of core-shell architectures with uniform shell thickness of ca. 3.5 nm. Moreover, the C-S NPC@Cu_2_O NNs show superior photocatalytic activity towards the photodegradation of MO with degradation rate as high as 6.67 mg min^−1^ g_cat_^−1^ under visible light irradiation, which can be attributed to the synergistic effects between unique core-shell nanostructure and 3D porous network with high specific surface area and fast mass transfer channel, indicative of a promising candidate for photocatalysts of MO degradation. This work will have important implications for facilely fabricating excellent core-shell architectured NPMs@M_x_O_y_ nanocomposites on a large scale towards practical applications of high-efficient, low-cost photocatalysts in photodegradation of organic dyes.

## Methods

### Mass synthesis of the C-S NPC@Cu_2_O NNs

Al-Cu alloy with nominal composition of 37 at.% Cu was prepared from pure Al (99.99 wt.%) and pure Cu (99.999 wt.%). Voltaic arc heating was employed to melt the charges in a copper crucible under an argon atmosphere, and then the melt was cooled down into ingots *in situ*. By use of a single roller melt spinning apparatus, the Al-Cu ingots were remelted in a quartz tube by high-frequency induction heating and then melt-spun onto a copper roller at a circumferential speed of ~3000 rpm in a controlled argon atmosphere. The Al-Cu alloy ribbons obtained were typically ca. 20 μm in thickness, 6–8 mm in width, and several centimeters in length. Energy dispersive X-ray (EDX) analysis showed the atomic percentage of Cu and Al in the initial Al-Cu alloy ribbons was quite closely to the designed composition, indicating the alloy ribbons can be further used in the following study (see Supplemental Information for EDX results, Table S1). Subsequently, the melt-spun Al-Cu alloy ribbons were chemically dealloyed in a 10 wt.% oxygen-rich NaOH aqueous solution at room temperature (RT, 20 °C) for 15 h to obtain the monolithic C-S NPC@Cu_2_O NNs. After the dealloying, the samples were rinsed with distilled water and dehydrated alcohol (analytical grade) several times, and then kept in a vacuum chamber to avoid oxidation further.

### Characterization of the C-S NPC@Cu_2_O NNs

Microstructural characterization and analysis of the melt-spun Al-Cu alloy ribbons and as-prepared nanocomposites were made using X-ray diffraction (XRD, Rigaku D/Max-2400) with Cu Kα radiation, scanning electron microscopy (FESEM, Hitachi S-4800) with an EDX analyzer, transmission electron microscopy (TEM, JEOL JEM 2100F). In order to evaluate specific surface area and pore size distribution of the as-prepared nanocomposites, the N_2_ adsorption/desorption experiments were carried out at 77 K on a Nova Station A automatic surface area and pore radius distribution apparatus.

To test the electrochemical activities of Al_2_Cu and AlCu phases in the Al 37 at.% Cu alloy, potentiodynamic polarization studies were conducted on single-phase Al_2_Cu and AlCu intermetallics (corresponding to Al 33 at.% Cu alloy and Al 50 at.% Cu alloy by the same preparation procedure mentioned above) in the oxygen-rich alkaline corrosive environment by using an electrochemical measurement unit (PARSTAT 2273). The experiments were carried out in a standard three-electrode electrochemical cell (200 mL) with a Pt plate electrode as a counter electrode, a saturated calomel electrode (SCE) as a reference electrode, and the alloy ribbon as the working electrode. Polarization scan was performed towards positive values at a scan rate of 1.0 mV s^−1^, after allowing a steady state potential to develop.

### Assessment of photocatalytic performance

To study the photocatalytic properties of the as-prepared nanocomposites towards the photodegradation of MO, the degradation experiment was carried out by 300 W xenon lamp (HSX-F300) for different durations. The detailed procedure is as follows: the as-prepared C-S NPC@Cu_2_O NNs (3 mg) was dispersed in a 100 mL beaker containing 90 mL MO aqueous solution (25 mg L^−1^), and was stirred in the dark for 20 min to achieve adsorption saturation. Then the suspension was exposed to 300 W Xe lamp (electric current: 15A) to carry out the photodegradation. The photodegradation experiment was performed under magnetic stirring conditions at ambient temperature. A small amount of MO solution (~4 mL) was extracted at time intervals of 20 min and the concentration of MO was monitored by an UV-vis spectrophotometer (Mapada) at wavelength of 465 nm, which is the maximum absorbance wavelength of MO. The photodegradation ratio (R) was calculated according to the following equation: R = (A_0_ − A)/A_0_ × 100%, where A_0_ is the original absorbance of MO at its maximum absorbance wavelength and A is the absorbance of MO at the same wavelength after different degradation durations.

## Additional Information

**How to cite this article**: Liu, W. *et al*. A facile one-pot oxidation-assisted dealloying protocol to massively synthesize monolithic core-shell architectured nanoporous copper@cuprous oxide nanonetworks for photodegradation of methyl orange. *Sci. Rep*. **6**, 36084; doi: 10.1038/srep36084 (2016).

**Publisher’s note**: Springer Nature remains neutral with regard to jurisdictional claims in published maps and institutional affiliations.

## Supplementary Material

Supplementary Information
