# Supplementary material for: A facile one-pot oxidation-assisted dealloying protocol to massively synthesize monolithic core-shell architectured nanoporous copper@cuprous oxide nanonetworks for photodegradation of methyl orange
Source: Sci Rep. 2016 Nov 10;6:36084. doi: 10.1038/srep36084 (PMC5103217; doi:10.1038/srep36084)
Supplement: Supplementary Information [file srep36084-s1.doc]

**Supplementary Information**

**A facile one-pot** **oxidation-assisted dealloying protocol to massively synthesize monolithic core-shell architectured nanoporous copper@cuprous oxide nanonetworks for photodegradation of methyl orange**

Wenbo Liu,^a,b,*^ Long Chen,^a^ Xin Dong,^a^ Jiazhen Yan,^a^ Ning Li,^a^ Sanqiang Shi,^b^ Shichao Zhang^c^

^a^ School of Manufacturing Science and Engineering, Sichuan University, Chengdu 610065, China

^b^ Department of Mechanical Engineering, The Hong Kong Polytechnic University,

Hung Hom, Kowloon, Hong Kong

^c^ School of Materials Science and Engineering, Beihang University, Beijing 100191, China

Tel: +86-028-85405320; Fax: +86-028-85403408; E-mail: liuwenbo_8338@163.com.

**Table S1.** Chemical composition of the initial Al-Cu alloy ribbons by EDX analysis.

| Specimens | Elements (at.%) | |
| --- | --- | --- |
|  | Al | Cu |
| Initial Al-Cu alloy ribbons | 63.18 | 36.82 |
